# Feasibility of a single-day protocol for SPECT and PET assessment of dopamine transporter availability, cardiac innervation and metabolic patterns in patients with movement disorders

**DOI:** 10.1007/s00259-025-07188-0

**Published:** 2025-03-11

**Authors:** Maximilian Scheifele, Johannes Gnörich, Elisabeth Schröder, Sophie C. Kunte, Zachary Ells, Johannes Hagen, Sabrina Katzdobler, Carla Palleis, Alexander Bernhardt, Alexander Jäck, Nicolai Franzmeier, Maximilian Fischer, Johannes Levin, Günter U. Höglinger, Rudolf A. Werner, Matthias Brendel

**Affiliations:** 1 Department of Nuclear Medicine, LMU University Hospital, LMU Munich, Munich, Germany; 2 German Center for Neurodegenerative Diseases (DZNE) Munich, Munich, Germany; 3 Department of Neurology, LMU University Hospital, LMU Munich, Munich, Germany; 4 Munich Cluster for Systems Neurology (SyNergy), Munich, Germany; 5 Ahmanson Translational Theranostics Division, Department of Molecular and Medical Pharmacology, University of California los Angeles UCLA, Los Angeles, CA U.S.A.; 6 Institute for Stroke and Dementia Research (ISD), LMU University Hospital, LMU Munich, Feodor-Lynen-Straße 17, 81377 Munich, Germany; 7 Institute of Neuroscience and Physiology, Department of Psychiatry and Neurochemistry, Mölndal and Gothenburg, University of Gothenburg, the Sahlgrenska Academy, Wallinsgatan 6, 431 41 Mölndal, Sweden; 8 Medizinische Klinik Und Poliklinik I, LMU University Hospital, LMU Munich, Marchioninistrasse 15, 81377 Munich, Germany; 9 DZHK (German Centre for Cardiovascular Research), Partner Site Munich Heart Alliance, Munich, Germany; 10 Russell H. Morgan Department of Radiology and Radiological Sciences, Johns Hopkins School of Medicine, Baltimore, MD U.S.A.; 11 BZKF (Bavarian Center for Cancer Research), Partner Site, Munich, Germany

**Keywords:** DaT imaging, Cardiac MIBG imaging, FDG-PET, Parkinson’s disease, Single day protocol

## Abstract

**Purpose:**

Due to new advances in molecular and imaging biomarkers, a biological classification of Parkinson’s disease (PD) called SyNeurGe (Hoglinger et al. Lancet Neurol 2024;23:191-204) has been proposed for research use recently. [^123^I]ioflupane dopamine transporter single-photon-emission-computed tomography (DaT-SPECT) and cardiac [^123^I]meta-iodobenzylguanidine (MIBG) scintigraphy are included in this biological classification scheme together with 2-[^18^F]fluoro-2-deoxy-D-glucose (FDG-PET) as central imaging biomarkers for the assessment of dopaminergic function, cardiac sympathetic denervation, and metabolic patterns in brain. In order to facilitate this prospectively high imaging demand and optimize diagnostic workup in PD we propose a single-day protocol.

**Methods:**

First, we excluded relevant binding of MIBG in the brain as well as DaT in the heart by acquisition of brain scans in patients that received MIBG as well as by acquisition of chest scans in patients that received DaT. Then, we performed a single-day protocol including DaT-SPECT and cardiac MIBG scintigraphy in ten patients with clinically suspected α-synucleinopathies (9 male, 1 female; 68.2 ± 7.3 years). Both radiotracers were injected simultaneously and cardiac imaging was performed at 3.5 h after injection followed by brain imaging at 4 h after injection using standard protocols for MIBG-scintigraphy and DaT-SPECT. Additionally, five patients of the dual tracer protocol group received brain FDG-PET after DaT and MIBG imaging.

**Results:**

Single tracer imaging confirmed no relevant uptake of [^123^I]ioflupane in the heart or [^123^I]MIBG in the brain. Six out of the ten dual tracer protocol patients (PD or multiple system atrophy with Parkinsonian phenotype (MSA-P)) showed a significantly reduced DaT-SPECT binding (z-score < -2) in at least one hemisphere (mean putaminal z-score -4.01 ± 1.39) while seven patients had a pathological heart-to-mediastinum ratio in the MIBG scan (mean H/M-ratio: 1.12 ± 0.08). Both DaT and MIBG scans could visually be interpreted without any signs of image artifacts or decrease in imaging quality and also quantitatively did not reveal significant differences to the single tracer scans. FDG-PET brain scans of the triple tracer protocol patients also showed no relevant interference in regard to image quality as well was generation of surface projections and z-scores.

**Conclusion:**

A single day protocol for DaT-SPECT, MIBG, and FDG-PET facilitates biomarker assessments needed for efficient biological characterization of Parkinsonian syndromes according to the SyNeurGe criteria.

**Supplementary Information:**

The online version contains supplementary material available at 10.1007/s00259-025-07188-0.

## Background

Parkinson's disease (PD) is a neurodegenerative disease characterized by a variety of motor and non-motor symptoms with misfolded neuronal a-synuclein as neuropathological hallmark for the definitive diagnosis [1, 2]. The complex pathophysiology presents significant challenges for diagnosis and classification. Due to recent biomarker and imaging advancements, biological classification schemes of PD and Dementia with Lewy bodies (DLB) have been proposed to enhance the current syndrome- and symptom-oriented classification [3, 4]. Along the seed amplification method for detecting pathological α-synuclein in cerebrospinal fluid (CSF) or immunohistochemical detection of α-synuclein in skin, imaging techniques such as [^123^I]ioflupane dopamine transporter single-photon-emission-computed tomography (DaT-SPECT) and cardiac [^123^I]meta-iodobenzylguanidine (MIBG) scintigraphy facilitate early and accurate diagnosis [5–7]. Together, these imaging methods offer a comprehensive assessment of central and peripheral nervous system involvement in PD. As they involve late imaging at 3 to 4 h post injection, these scans are usually obtained on separate days at nuclear medicine facilities. However, as both modalities share I-123 as radionuclide, i.e. are measured in the same energy window, they yet bind to different target regions, such that a dual-tracer protocol could be feasible. This study explores the integration of both imaging modalities using a dual injection, single-day protocol with the potential addition of an 2-[^18^F]fluoro-2-deoxy-D-glucose (FDG-PET) brain scan. We evaluate if such a protocol is technically feasible to fulfill the rising demand of examinations, as expected by the increasing use of biological classification schemes of PD. Thus, a single-day protocol would highly increase efficiency of the diagnostic workflow as well as patient comfort.

## Methods

### Patient cohort and imaging

To exclude relevant binding of [^123^I]MIBG in the brain as well as [^123^I]ioflupane in the heart 13 patients with suspected α-synucleinopathies (Parkinsons Disease (PD), Multiple system atrophy (MSA), Lewy body dementia (LBD)) underwent eihter a DaT-SPECT, including a quantitative SPECT of the thorax (n = 7, 4 male, 67.1 ± 9.7 years), or a MIBG-scintigraphy which included a quantitative SPECT of the brain (n = 6, 3 male, 55.5 ± 12.9 years). Additionally, ten patients with clinically diagnosed α-synucleinopathies (PD, Rapid eye movement sleep behavior disorder (RBD), MSA) were injected simultaneously with [^123^I]ioflupane and [^123^I]MIBG (9 male, 1 female; 68.2 ± 7.3 years). 3.5 h after injection MIBG planar scintigraphies of the thorax were performed on a SPECT/CT system (Mediso Anyscan Trio, Budapest Hungary) using standard protocols for MIBG imaging. 4 h after injection [^123^I]ioflupane brain SPECT was performed using the same camera and standard protocol for DaT imaging [8]. SPECT and SPECT/CT imaging was performed using a low energy collimator. Images were reconstructed iteratively including a monte carlo based scatter correction as well as a ldCT based attenuation correction when applicable. The injected dose for both [^123^I]ioflupane and [^123^I]MIBG was 185 MBq. Five of these patients additionally received an FDG-PET brain scan on the same day after both [^123^I] measurements using the EANM standard protocol with a dose of 150 MBq [^18^F]-FDG [9] (Siemens Biograph, Erlangen Germany). All patients received perchlorate thirty minutes prior to the injection in order to inhibit [^123^I] uptake by the thyroid. All participants provided written informed consent for the imaging procedures. The study was conducted in accordance with the principles of the Declaration of Helsinki. Approval for scientific data analysis was obtained from the local ethics committee (application number: 21–0721).

### Image analysis

DaT-SPECT scans were examined using Hermes BRASS (Hermes Medical Solutions, Stockholm, Sweden, BRASS model 5) and GE DaTQuant (GE HealthCare, Chicago, United States, GE DaTQuant version 2.0) with the occipital lobe as reference region. Caudate nucleus, anterior putamen and posterior putamen (all bilateral) were designated as target regions. Z-scores, which represent the number of standard deviations a value deviates from the mean of a normative population, were calculated for these regions after age correction, providing a standardized metric for identifying abnormalities. A z-score below −2 was considered pathological. For examination of the MIBG scans heart-to-mediastinum ratios (H/M-ratio) were obtained using mean count rates of standardized circular and rectangular regions of interest (ROI) for the heart and the mediastinum according to the imaging guidelines for SPECT nuclear cardiology procedures of the american society of nuclear cardiology (ASNC) [10]. A H/M-ratio of 1.7 was set as cut-off value [11]. As a semi-quantitative measure, we also obtained standard uptake values (SUV) by quantifying radiotracer uptake in standardized brain (striatum, occipital lobe, whole brain) and mediastinum regions of interest relative to the injected dose and body weight of the patient [12].

FDG-PET scans were analyzed using 3D stereotactic surface projections (3DSSP) to visualize and quantify metabolic patterns across the brain. Surface projections were generated to compare regional brain metabolism with a normative database using z-scores.

### Statistics

This study was designed as a proof-of-concept investigation to assess the feasibility of a single-day imaging protocol. Given the technical complexity and the need to verify potential tracer interactions, a limited cohort was selected. For the single tracer group comparisons of cardiac tracer uptake SUV values (SUVmean) of the heart and H/M-ratios were compared i) after [^123^I]ioflupane injection, ii) after [^123^I]MIBG injection and visually normal cardiac uptake, and iii) after [^123^I]MIBG injection and visually abnormal cardiac uptake using a one-way ANOVA with Tukey post hoc correction for multiple comparisons. For striatal tracer uptake SUV values (SUVmax) of the basal ganglia were compared i) after [^123^I]MIBG injection, ii) after [^123^I]ioflupane injection and visually normal basal ganglia uptake, and iii) after [^123^I]ioflupane injection and visually abnormal basal ganglia uptake using a one-way ANOVA with Tukey post hoc correction for multiple comparisons. To compare group means in SUV between dual and single tracer studies we used an unpaired t-test. Due to the limited anatomical resolution of SPECT imaging, SUVmax was chosen for striatal analysis to enhance sensitivity for detecting potential MIBG uptake in the basal ganglia.

For the ten patients with a single day protocol of DaT-SPECT and MIBG, z-scores and striatal binding ratios (SBR) of DaT expression were quantified using two independent software packages, Hermes BRASS and GE DaTQuant. The correlation between these values was analyzed using simple linear regression, allowing for cross-validation of automated quantification methods.

## Results

### Exclusion of relevant cardiac tracer uptake in the DaT-SPECT group

**Fig. 1:**
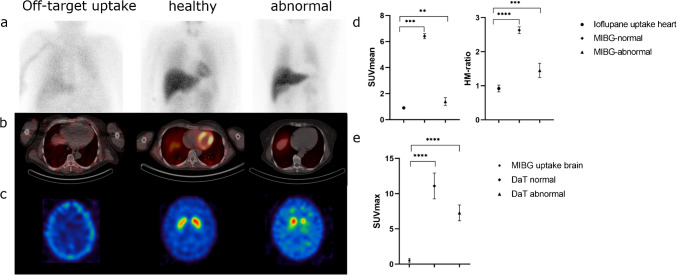
Comparison of single tracer scans. **(a)** planar scintigraphies (anterior view) of the chest of a patient injected with [^123^I]ioflupane as well as a normal and abnormal scan of patients injected with [^123^I]MIBG (left to right)**. (b)** corresponding axial fused cardiac SPECT/CT-images of the patients depicted in row a**. (c)** axial brain SPECT images of a patient injected with [^123^I]MIBG as well as a normal and abnormal scan of patients injected with [^123^I]ioflupane (left to right). **(d)** quantitative and semi-quantitative (heart-to-mediastinum ratio, HM-ratio) comparison of cardiac tracer signals after i) after [^123^I]ioflupane injection, ii) after [^123^I]MIBG injection and visually normal cardiac uptake, and iii) after [^123^I]MIBG injection and visually abnormal cardiac uptake using a one-way ANOVA with Tukey post hoc correction for multiple comparisons **(e)** quantitative comparison of tracer signals in the basal ganglia after i) [^123^I]MIBG injection, ii) [^123^I]ioflupane injection and visually normal basal ganglia uptake, and iii) [^123^I]ioflupane injection and visually abnormal basal ganglia uptake using a one-way ANOVA with Tukey post hoc correction for multiple comparisons

Visual and quantitative assessment revealed no relevant uptake of [^123^I]ioflupane in the myocardium. Patients who only received a DaT-SPECT (n = 7, 4 male, 67.1 ± 9.7 years) and underwent an additional SPECT of the thorax presented a mean SUV of 0.91 ± 0.07 in the myocardium and a mean H/M-ratio of 0.92 ± 0.10 (see Fig. 1). Five out of the seven patients had a significantly reduced striatal DaT availability (z-score < −2) in at least one hemisphere. Quantitatively, patients of the MIBG group with an unimpaired cardiac sympathetic innervation (n = 4) presented with a mean SUV of 6.41 ± 0.19, while patients with a reduced cardiac MIBG uptake (n = 2) showed a mean SUV of 1.40 ± 0.30, still exceeding the quantitative uptake after DaT injection (p = 0.0060, see Fig. 1d).

### Exclusion of relevant striatal tracer uptake in the MIBG group

Visual and quantitative assessment revealed no relevant uptake of [^123^I]-MIBG in the striatum or the occipital region. Brain SPECT acquisitions in the MIBG group (n = 6, 3 male, 55.5 ± 12.9 years) showed a mean SUV of 0.15 ± 0.06 in the striatum (see Fig. 1e). Two patients of this group showed reduced cardiac MIBG uptake below the cut-off H/M-ratio of 1.7. In contrast, the DaT-SPECT group patients with a normal striatal tracer uptake showed a maximum SUV of 11.1 ± 1.80 (n = 2) while the patients with an abnormal scan showed a maximum SUV of 7.28 ± 1.13 (n = 5; see Fig. 1e), both significantly exceeding the quantitative MIBG signal in the striatum (both p < 0.001).

### Dual tracer application provides robust assessment of DaT and MIBG

**Fig. 2:**
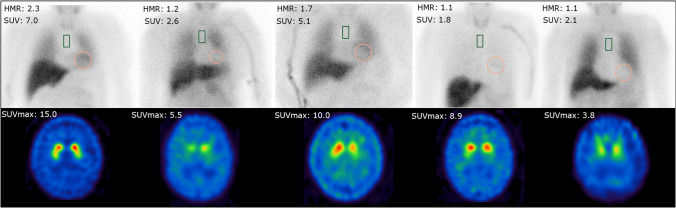
Dual tracer group. Planar [^123^I]MIBG scans including heart-to-mediastinum ratio and mean myocardial SUV value from quantitative SPECT/CT images (upper row) and the corresponding [^123^I]ioflupane SPECT images including striatal SUVmax values of the 5 patients that received a dual tracer protocol

Ten patients were simultaneously injected with [^123^I]ioflupane and [^123^I]-MIBG (9 male, 1 female; 68.2 ± 7.3 years). Six out of the ten patients showed a pathological DaT-SPECT result in at least one hemisphere with a mean putaminal z-score of −4.01 ± 1.39 while four patients had a normal result with a mean putaminal z-score of −0.79 ± 1.01. In regard to the MIBG scans seven patients showed a reduced cardiac MIBG uptake with a mean SUV of 2.79 ± 0.92 and a mean H/M-ratio of 1.11 ± 0.04 while three patients had a normal cardiac MIBG uptake with a mean SUV of 6.37 ± 1.10 and a H/M-ratio of 1.95 ± 0.31. Exemplary images of five patients are shown in Fig. 2.

Comparing the mean of measured SUV of dual tracer versus single tracer acquisitions using an unpaired t-test did not reveal significant differences regarding the SUVmean for patients with normal (n = 3 + 4, p = 0.977) and abnormal (n = 7 + 2, p = 0.084) MIBG uptake, as well as for the SUVmax in normal (n = 4 + 2, p = 0.318) and abnormal (n = 6 + 5, p = 0.367) DaT availability. Visually some of the dual tracer DaT-SPECT scans showed a slightly elevated tracer uptake within the skull when compared to single tracer DaT-SPECT acquisitions (for example see Fig. 2, case 3).

**Fig. 3:**
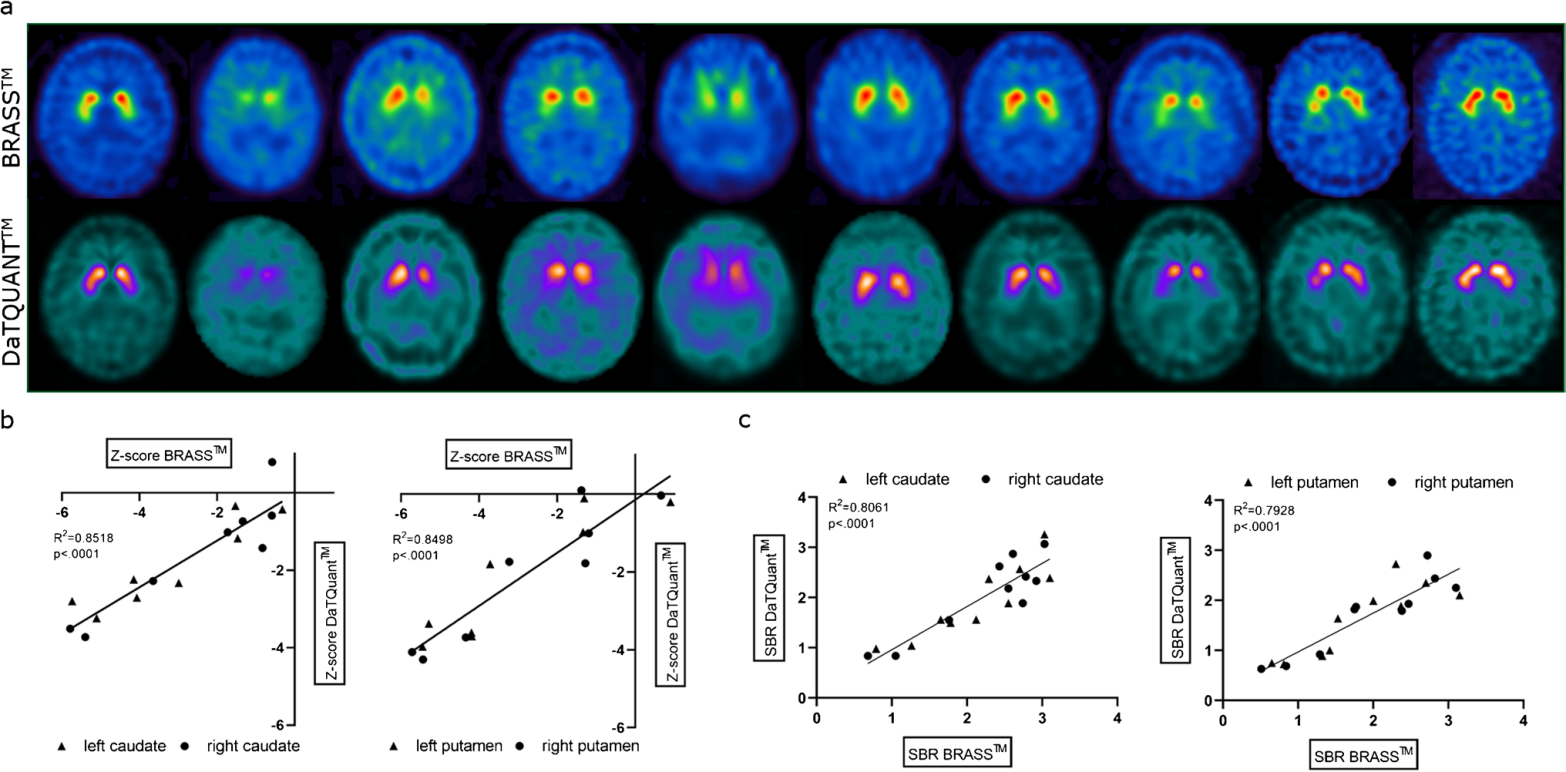
Comparison of results from different software tools for single day protocol group 123I-Ioflupane-SPECT scans.** (a)** [^123^I]ioflupane SPECT scans visualized in Hermes BRASS™ (upper row) and GE DaTQuant^Tm^ (lower row). **(b)** results from linear regression correlating z-score values generated by Hermes BRASS™ (x-axis) and GE DaTQuant™(y-axis) from caudate and putamen including corresponding R^2^ and p-values. **(c)** results from linear regression correlating specific binding ratio (SBR) values generated by Hermes BRASS™ (x-axis) and GE DaTQuant™ (y-axis) from caudate and putamen including corresponding R^2^ and p-values

We tested two quantitative software products (Hermes BRASS and GE DaTQuant) to see if there would be any interference with the detection of the specific binding ratio (SBR) and z-score generation, i.e. to test if the slight skull uptake hampers automated processing of the scans. Both software packages were able to generate z-scores from the scans and a simple regression analysis revealed a strong correlation between the SBR and z-scores from both software packages (see Fig. 3) with R^2^ = 0.780 for z-scores of the caudate (p < 0.0001), R^2^ = 0.806 for the SBR of the caudate (p < 0.0001) and R^2^ = 0.767 for z-scores of the putamen (p < 0.0001) as well as R^2^ = 0.793 for the SBR of the putamen (p < 0.0001). In the supplemental information we also added two figures in which we adjusted the SUV in the brain as well as the mediastinal and cardiac count rates of the dual tracer scans for the possible added background signal introduced by dual tracer injections. SUV ratios between the striatal and occipital region in the dual tracer scans were not affected by the background correction (see supplemental Fig. 1a). The H/M-ratios showed a slight increase after the background correction but all patients below the threshold of 1.7 stayed below this mark (see supplemental Fig. 1b).

### A triple tracer protocol allows assessment of striatal DaT, cardiac MIBG and brain metabolic patterns on a single day

**Fig. 4:**
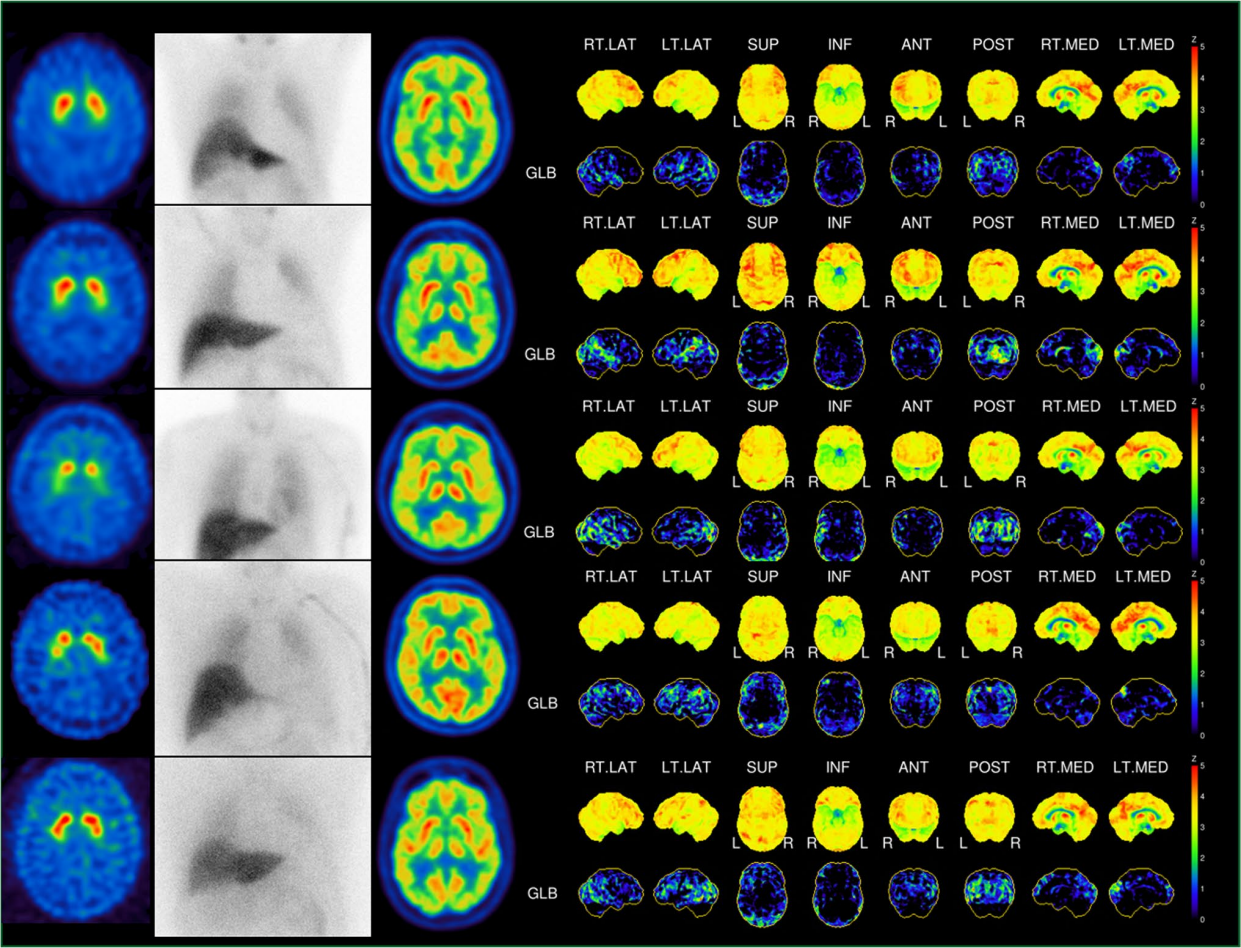
Triple tracer group. Overview of the five patients that received a triple tracer injection on a single day depicting the corresponding [^123^I]ioflupane SPECT, planar MIBG scan, axial FDG brain PET as well as FDG surface projections with z-scores

**Fig. 5:**
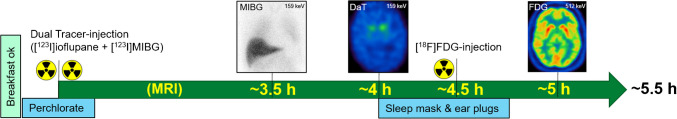
Timeline triple tracer protocol. Visualization of the timeline for a single day triple tracer protocol with planar MIBG scan, Ioflupane SPECT and axial FDG brain PET of an example case. Patients can have a breakfast in the morning and receive Perchlorate 30 min prior to the dual tracer injection of [^123^I]ioflupane + [^123^I]MIBG. After dual tracer injection additional an MRI scan could be feasible. 3.5 h MIBG imaging begins and is followed by DaT imaging at 4 h post injection. During DaT imaging the patient can wear a sleep mask and ear plugs in preparation for the following FDG brain PET. After DaT imaging [^18^F]FDG then can be injected at around 4.5 h after the dual tracer injection and PET imaging can start 30 min later

Five patients of the dual tracer group (5 male, 67.4 ± 7.32 years) received an additional [^18^F]FDG brain PET following DaT and MIBG imaging as a triple tracer protocol. Upon visual inspection, there was no relevant interference of dual tracer SPECT imaging before the [^18^F]FDG scan. Surface projections and z-score calculations could be generated without restrictions (see Fig. 4). The timeline of the proposed single day triple tracer protocol is visualized in Fig. 5.

## Discussion

The present study proposes a single-day protocol utilizing dual-injection [^123^I]ioflupane SPECT and [^123^I]MIBG scintigraphy for the evaluation of Parkinson’s disease (PD) patients with optional subsequent assessment of [^18^F]FDG-PET (see Fig. 5). Aligning with the recently proposed biological classification schemes of PD, such as the SyNeurGe classification [3], this combined approach can help streamline the diagnostic workflow while maintaining diagnostic accuracy.

The rationale behind the proposed single day protocol is founded on the assumption that there is no interference regarding binding properties of Ioflupane and MIBG while sharing the same energy peak of I-123 at 159 keV. This energy window subsequently allows an additional [^18^F]FDG brain PET scan that uses a much higher energy window of 511 keV excluding interference of the previous I-123 scans. Ioflupane on the one hand is a well-established radiotracer that shows a high binding affinity for the presynaptic dopamine transporter in striatal neurons [13–15]. DaT is expressed almost exclusively in the dopaminergic neurons of the midbrain that project into the striatum and there is no evident DaT expression in cardiomyocytes [16–18]. MIBG on the other hand is a norepinephrine analog that under healthy conditions is not able to pass the blood brain barrier and therefore should show no relevant binding to striatal structures [19–21]. These assumptions are approved by the results of our work which in regard to H/M-ratios and/or SUV values indicate no relevant binding of Ioflupane in the heart as well as MIBG in the brain (see Fig. 1).

However several limitations should be acknowledged. This study was conducted on a relatively small cohort of 10 patients who underwent the dual-tracer protocol, with only five receiving the triple-tracer protocol. Patients were diagnosed with clinically suspected α-synucleinopathies, which may introduce selection bias and the limited sample size restricts the generalizability of the findings. We emphasize that the present study was designed as a proof-of-concept investigation to demonstrate the feasibility of the single-day imaging protocol. In future, expanding the sample size across multiple centers with diverse patient populations will help confirm the reliability of the single-day protocol and validate our findings.

Regarding MIBG it has to be considered that in patients with rare conditions that compromise blood brain barrier function an increased tracer uptake within the brain could be possible [22]. For such patients (i.e. an unclear case of Creutzfeld-Jakob-disease) separate imaging appointments should be preferred. Another possible interference in regard to MIBG might be the slightly increased skull uptake which we could detect in few cases (see Fig. 2, case 3). To address this issue, we used two different software packages for SBR calculation and z-score generation in Ioflupane SPECT and both were able to generate SBR values and z-scores from the acquired images with significantly correlating values (see Fig. 4).

The concurrent use of two [^123^I]-labeled tracers introduces potential challenges related to the interference of image analysis due to added background signal as well as the timing of imaging acquisition. For the quantitative analysis of DAT binding the extremely low background signal of MIBG should have no significant effect. In regard to assessment of the MIBG scans the added background signal possibly leads to a slight reduction of the H/M-ratios which needs to be considered in borderline cases (see supplemental Fig. 1b). Future studies with bigger sample sizes are needed to validate if there needs to be an adjustment to the H/M-ratio threshold. Another way to overcome this possible influence would be to use myocardial SUV measurements by quantitative SPECT/CT rather than the 2-dimensional H/M-ratio. This three-dimensional approach to a three-dimensional structure could increase diagnostic accuracy. In a recent [123I]-MIBG study we could already show that the use of quantitative myocardial SUV provides stronger discrimination between patients of the Parkinson’s disease continuum and patients with Arrhythmogenic Right Ventricular Cardiomyopathy compared to the H/M-ratio [23].

In regard to acquisition timing, we opted for starting with MIBG scintigraphy at 3.5 h immediately with a scan duration of 30 min directly followed by the DaT-SPECT at around 4 h post injection as our scanner specific normal population for Ioflupane SPECT was scanned at 4 h post injection (see Fig. 5). Imaging windows for both tracers are within 3–6 h after injection. While DAT binding is stable at 3–6 h post injection [24, 25] optimal MIBG scanning time in heart failure patients is usually recommended at 4 h post injection [26]. However, in regard to PD diagnosis earlier timepoints up to 2 h post injection also showed accurate diagnostic performance [27, 28]. In this perspective variations of the protocol should also be feasible (i.e. starting with a Ioflupane SPECT at 3 h p.i. followed by the MIBG scan) and additional imaging techniques such as diffusion tensor imaging MRI (DTI-MRI) could be implemented.

From a patient’s point of view, a single day protocol would improve convenience and possibly lead to a higher patient compliance. Otherwise, patients would need to return on multiple days for separate imaging sessions. Especially for those with mobility issues or those traveling from distant locations a single-day protocol can mitigate these challenges.

## Conclusions

Our findings indicate that the single-day protocol of striatal DaT, cardiac MIBG and metabolic pattern assessment is both feasible and effective. Integrating these modalities into a single session reduces the overall time required for a comprehensive diagnostic evaluation without compromising the quality of the results and therefore offers a possibility to fulfill the prospectively rapidly rising demand of examinations in the new age of a biomarker-based classification of Parkinson’s disease.

## Supplementary Information

Below is the link to the electronic supplementary material.

Supplementary file1 (DOCX 240 KB)

## Data Availability

Relevant data generated or analyzed during this study are included in this published article. Further datasets used and/or analyzed during the current study are available from the corresponding author upon reasonable request.
