# Supplementary material for: Feasibility of a single-day protocol for SPECT and PET assessment of dopamine transporter availability, cardiac innervation and metabolic patterns in patients with movement disorders
Source: Eur J Nucl Med Mol Imaging. 2025 Mar 11;52(9):3424–32. doi: 10.1007/s00259-025-07188-0 (PMC12222253; doi:10.1007/s00259-025-07188-0)
Supplement: Supplementary file 1 — Supplementary file1 (DOCX 240 KB) [file 259_2025_7188_MOESM1_ESM.docx]

**Supplementary Information**

**Feasibility of a single-day protocol for SPECT and PET assessment of dopamine transporter availability, cardiac innervation and metabolic patterns in patients with movement disorders**

Maximilian Scheifele^1^, Johannes Gnörich^1^, Elisabeth Schröder^1^, Sophie C. Kunte^1,11^, Zachary Ells^1,5^, Johannes Hagen^1^, Sabrina Katzdobler^2,3,4^, Carla Palleis^2,3,4^, Alexander Bernhardt^3^, Alexander Jäck^3^, Nicolai Franzmeier^4,6,7^, Maximilian Fischer^8,9^, Johannes Levin^2,3,4^, Günter U. Höglinger^2,3,4^, Rudolf A. Werner^1,10^, Matthias Brendel^1,2,4^

^1^Department of Nuclear Medicine, LMU University Hospital, LMU Munich, Munich, Germany

^2^German Center for Neurodegenerative Diseases (DZNE) Munich, Munich, Germany

^3^Department of Neurology, LMU University Hospital, LMU Munich, Munich, Germany

^4^Munich Cluster for Systems Neurology (SyNergy), Munich, Germany

^5^Ahmanson Translational Theranostics Division, Department of Molecular and Medical Pharmacology, University of California Los Angeles UCLA, Los Angeles, California, USA

^6^Institute for Stroke and Dementia Research (ISD), LMU University Hospital, LMU Munich, Feodor-Lynen-Straße 17, 81377 Munich, Germany

^7^University of Gothenburg, The Sahlgrenska Academy, Institute of Neuroscience and Physiology, Department of Psychiatry and Neurochemistry, Mölndal and Gothenburg, Wallinsgatan 6, 431 41 Mölndal, Sweden

^8^Medizinische Klinik und Poliklinik I, LMU University Hospital, LMU Munich, Marchioninistrasse 15, 81377 Munich, Germany

^9^DZHK (German Centre for Cardiovascular Research), Partner Site Munich Heart Alliance, Munich, Germany

^10^Russell H. Morgan Department of Radiology and Radiological Sciences, Johns Hopkins School of Medicine, Baltimore, Maryland

^11^BZKF (Bavarian Center for Cancer Research), Partner site Munich, Germany

**Corresponding author:**

Maximilian Scheifele

Department of Nuclear Medicine

LMU University Hospital, LMU Munich, Munich, Germany

Phone: +49 (0) 89 4400 74646

E-Mail: Maximilian.Scheifele@med.uni-muenchen.de

**Supplemental Figure 1: Background correction for dual tracer scans**


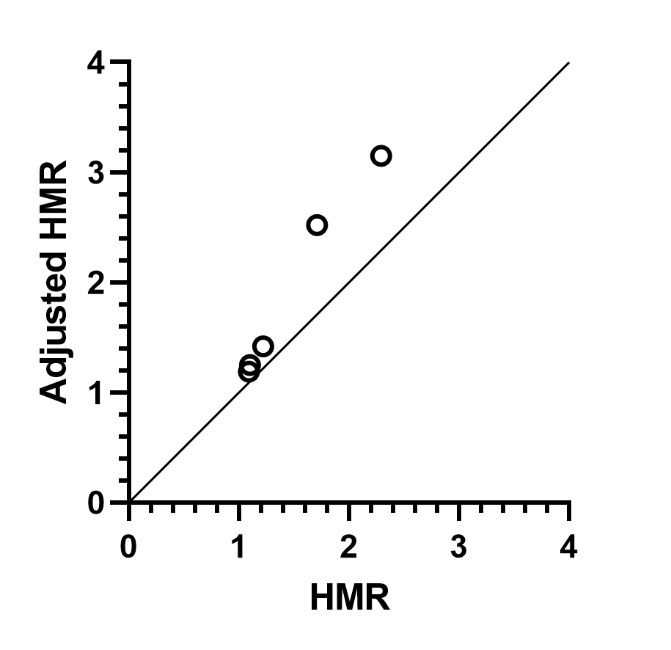

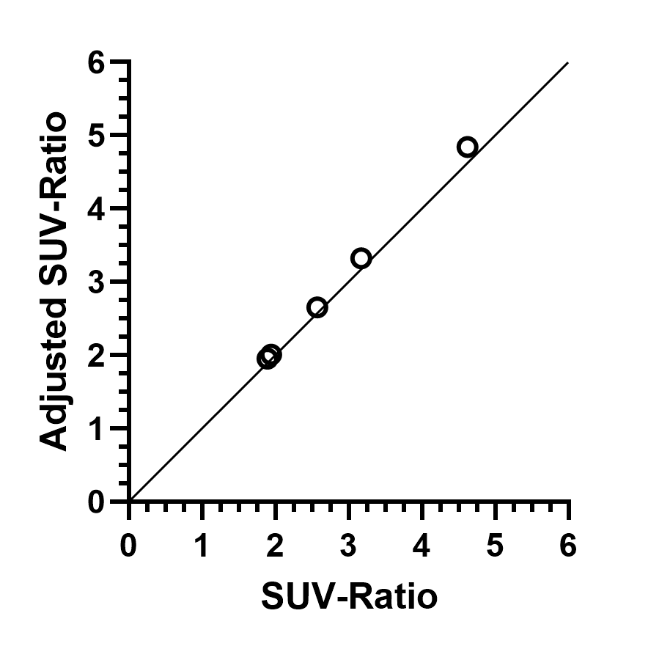


a

b

**Supplemental Figure 1:**

(a) X-axis indicates the SUVmean-Ratio between the left striatum and the occipital lobe measured in the dual tracer scans. Y-axis indicates the same SUV-Ratio adjusted for the background signal of MIBG. Line of identity is included to visualize the impact of added background signal for dual tracer scans.

(b) ) X-axis indicates the H/M-ratio measured in dual tracer scans. Y-axis indicates the H/M-ratio adjusted for the background signal of Ioflupane. Line of identity is included to visualize the impact of added background signal for dual tracer scans. Red bars indicate the threshold of 1.7.
